# Diagnosis of axillary nodal metastases by ultrasound-guided core biopsy in primary operable breast cancer

**DOI:** 10.1038/sj.bjc.6601290

**Published:** 2003-09-30

**Authors:** A Damera, A J Evans, E J Cornford, A R M Wilson, H C Burrell, J J James, S E Pinder, I O Ellis, A H S Lee, R D Macmillan

**Affiliations:** 1 Department of Radiology, Helen Garrod Breast Screening Unit, Nottingham International Breast Education Centre, Nottingham City Hospital, Hucknall Road, Nottingham NG5 1PB, UK; 2 Department of Histopathology, Nottingham City Hospital, Nottingham NG5 1PB, UK; 3 Department of Breast Surgery, Nottingham City Hospital, Nottingham NG5 1PB, UK

**Keywords:** ultrasound, core biopsy, breast tumour, axilla

## Abstract

The purpose of this study was to examine the use of ultrasound (US)-guided core biopsy of axillary nodes in patients with operable breast cancer. The ipsilateral axillae of 187 patients with suspected primary operable breast cancer were scanned. Nodes were classified based on their shape and cortical morphology. Abnormal nodes underwent US-guided core biopsy/fine needle aspiration (FNA), and the results correlated with subsequent axillary surgery. The nodes were identified on US in 103 of 166 axillae of patients with confirmed invasive carcinoma. In total, 54 (52%) met the criteria for biopsy: 48 core biopsies (26 malignant, 20 benign node, two normal) and six FNA were performed. On subsequent definitive histological examination, 64 of 166 (39%) had axillary metastases. Of the 64 patients with involved nodes at surgery, preoperative US identified nodes in 46 patients (72%), of which 35 (55%) met the criteria for biopsy and 27 (42%) of these were diagnosed preoperatively by US-guided biopsy. In conclusion, US can identify abnormal nodes in patients presenting with primary operable breast cancer. In all, 65% of these nodes are malignant and this can often be confirmed with US-guided core biopsy.

The primary prognostic discriminant in patients presenting with breast cancer is lymph node status (Fisher *et al*, 1983). Traditionally, axillary dissection at the time of mastectomy or breast conservation surgery is the method for obtaining this information regarding the histological nodal status of the patient. Axillary dissection is commonly associated with morbidity (Ivens *et al*, 1992; Duff *et al*, 2001), and patients who are node negative derive no benefit from it, hence the recent interest in less invasive staging procedures such as sentinel node biopsy and axillary node sampling. The aim of imaging would be to identify patients with nodal metastases for whom axillary node clearance would be appropriate. Other patients at low risk of nodal metastases would be selected for either sampling or sentinel node biopsy.

Existing nonsurgical techniques for preoperative axillary staging include clinical examination, ultrasonography (US), colour Doppler, scintimammography, high-resolution computed tomography (HRCT), dynamic contrast enhanced magnetic resonance imaging (MRI) and positron emission tomography (PET). The range of sensitivity and specificity quoted in the literature for these techniques is such that the clinical management of the axilla cannot be modified for individual patients based upon these test results. US-guided fine needle aspiration (FNA) has been able to identify 36 – 63% of node-positive patients preoperatively (Bonnema *et al*, 1997; De Kanter *et al*, 1999). Image-guided core biopsy has a higher sensitivity than FNA for assessing breast lesions (Britton and McCann, 1999), and therefore it would be anticipated that a similar improvement in sensitivity would be observed in US-guided core biopsy of the axilla.

The purpose of this study was to examine the usefulness of ultrasound (US)-guided core biopsy of abnormal axillary nodes in patients presenting with primary operable breast cancer to enable level 3 clearance of patients with histologically confirmed nodal metastases. Patients without histologically confirmed nodal metastases would then benefit from the lower morbidity of sampling/sentinel node procedures and subsequent radiotherapy, where preoperative core biopsy results were falsely negative. To our knowledge, this is the first study to use 14-gauge core biopsy to obtain axillary node samples in this clinical setting.

## MATERIALS AND METHODS

Over a 9-month period between February 2002 and November 2002, patients who presented with suspected operable, invasive breast cancer had their ipsilateral axilla scanned routinely. US assessment of the axilla was carried out using an 8 – 14 MHz probe (Esoate, Technos 447Zi plus) at the same time as a US scan of the suspected breast malignant lesion. The axillae were carefully scanned with particular attention to the lower axilla, posterior to where the pectoralis major muscle crosses the cranial edge of the breast disc, a common location for the sentinel node (Krag *et al*, 1998).

Nodal features assessed were the shape and the morphology of the cortex. Nodes were categorised as abnormal in shape if the longitudinal to the transverse axis ratio was less than two. Nodes were considered as having abnormal morphology if the cortex was concentrically or eccentrically thickened to more than 2 mm. A US-guided core biopsy using a 14-gauge needle was performed on abnormal nodes. When more than one abnormal node was identified in the axilla, the node that looked most abnormal was biopsied. Whenever possible, an average of two cores was obtained. When it was technically difficult to perform a core biopsy due to the proximity of axillary vessels, a US-guided FNA was carried out. The core biopsy results were categorised as benign node, malignant node and normal, when no nodal tissue was identified in the sample. FNA results in our unit are categorised as insufficient, benign, equivocal, suspicious and malignant.

All the patients who had nodal metastases on core biopsy underwent level 3 axillary clearance as a primary procedure at the time of resection of the primary tumour. The lymph node stage was determined by the number of positive nodes on the histology of the surgical specimen; lymph node stage 1 if no nodes were involved, lymph node stage 2 if three or less nodes were involved and lymph node stage 3 for four or more involved nodes. Those patients who did not have nodal metastases confirmed histologically preoperatively underwent axillary node sampling with or without sentinel node augmentation, depending on the surgical preference. Those patients who were shown to have axillary nodal metastases subsequently underwent either radiotherapy if stage 2, or axillary clearance if stage 3.

The size and grade of the primary tumour were documented. A note was made of the mode of presentation of the breast cancer, that is, whether screen detected or symptomatic, and also if the axillary nodes were clinically palpable or not. At the end of the study period, all patients having lesions with clinical or imaging features suspicious of malignancy, but with benign pathology on core biopsy or surgery, were excluded from the study group. Patients were excluded if they did not proceed to surgery because of a variety of reasons, the most common being medical unfitness for surgery. Patients with definite locally advanced disease were also excluded, as they would not have immediate breast or axillary surgery.

## RESULTS

Of the 187 patients whose axillae were scanned, 166 with invasive cancer were included in the study group. The age range was 33 – 81 years (median age 56 years). The tumour types encountered in our study group were ductal NST in 92 patients (55%), tubular mixed in 35 (21%), mixed ductal/lobular in 15 (9%), lobular classical in 10 (6%), tubular in three (2%) and other types in 11 (7%). Of the 166 patients, 10 (6%) had clinically palpable nodes suspicious of metastatic disease. Eight of the 10 patients with clinically palpable nodes had metastases. All eight had more than three involved nodes at surgery. In total, 55 (33%) of the cancers were screen detected and 111 (67%) presented symptomatically. Nodes were identified on US in 103 (62%) of 166 axillae scanned. Of these, 54 (33%) met the criteria for biopsy. In total, 48 core biopsies and six FNAs were performed. Of the 48 core biopsies, 26 were malignant, 20 were benign nodes and two normal. Of the six FNAs, one was benign, four inadequate and one suspicious for malignancy. There were no complications following core biopsy of the axillary nodes and our technique did not affect the subsequent axillary surgical procedure.

In total, 64 (39%) of 166 patients had axillary metastases at surgery. US-guided core biopsy/FNA identified 27 (42%) of 64 node-positive patients preoperatively. Analysis of our false negative results revealed that in 18 patients nodes were not visualised, in 11 patients nodes were identified but did not fit our criteria for biopsy, in seven patients the core biopsy results were benign and in one patient the FNA sample was insufficient for diagnosis.

**Figure 1:**
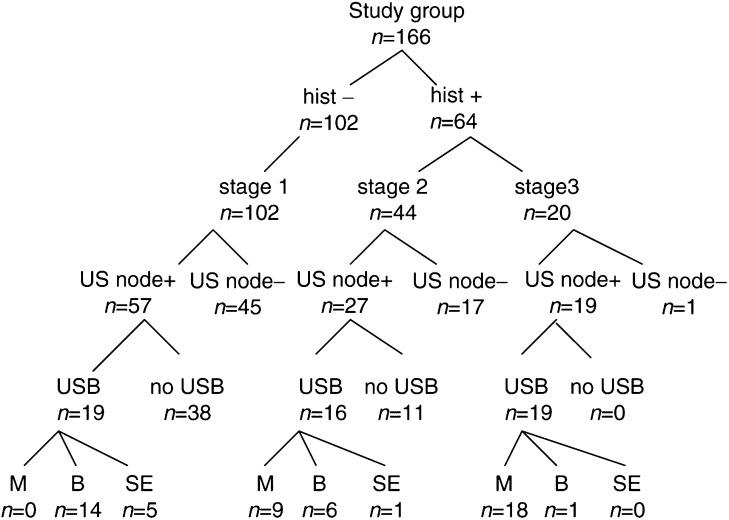
Surgical histology and preoperative US core biopsy results. *n*=no. of patients; hist−=no malignant nodes on surgical histology; hist+=malignant nodes on surgical histology; stage 1=no positive nodes; stage 2=1 – 3 positive nodes; stage 3=4 or more positive nodes; US node+=nodes seen on US; US node−=no nodes seen on US; USB=US core biopsy performed; no USB=US core biopsy not carried out as nodes did not meet our criteria for biopsy: M=malignant; B=benign; SE=sampling error.

Preoperative US findings and the results of US-guided core biopsies when correlated with the nodal stage at surgery are as shown in Figure 1

. The detection of abnormal nodes on US biopsy increased with increasing burden of metastatic disease in the axilla. In the 20 patients with more than three involved nodes, 18 (90%) were diagnosed as node positive preoperatively. In contrast, US-guided biopsy detected only nine (20%) of 44 patients with three or less involved nodes at surgery.

US had a sensitivity of 55%, a specificity of 82%, a positive predictive value (PPV) of 74% and a negative predictive value (NPV) of 65% in identifying nodal metastases. The overall sensitivity, specificity, PPV and NPV of US-guided biopsy was 42, 100, 100 and 74%, respectively. In our study, the nodal positivity rates in patients with no nodes, normal nodes and abnormal nodes seen on US were 29% (18 of 63), 22% (11 of 49) and 65% (35 of 54), respectively. The size and grade of the invasive carcinoma influences the incidence of axillary metastases and their detection preoperatively by US-guided biopsy (Tables 1

**Table 1:** Correlation of tumour grade and node positivity

**Grade of the tumour**	**No. of patients**	**Node positivity at surgery**	**Node positivity on US biopsy**
Grade 1	37	9 (24%)	2 (22%)
Grade 2	64	23 (36%)	4 (17%)
Grade 3	65	32 (49%)	21 (66%)

and 2

**Table 2:** Correlation of invasive cancer size and node positivity

**Size of invasive cancer at histology (mm)**	**No. of patients**	**Node positivity at surgery (%)**	**Node positivity on US biopsy (%)**
<10	17	2 (12%)	1 (50%)
10 – 14	39	10 (26%)	2 (20%)
15 – 19	42	16 (38%)	4 (25%)
20 – 29	45	19 (42%)	10 (53%)
>30	23	17 (74%)	10 (59%)
Total no.	166	64 (39%)	27 (42%)

In 17 patients, the primary tumour was multifocal. The sum of the sizes of all invasive foci was taken as the final measurement.

). In grade 1 tumours, the likelihood of detecting involved nodes preoperatively was low (22%) when compared to detection in grade 3 tumours (66%). When the size of the primary tumour was less than 15 mm, the incidence of metastases was 21% (12 of 56) and we could only detect 25% (three of 12) of node-positive patients preoperatively. However, when the size of the primary tumour was more than 15 mm, the incidence of axillary metastases was 47% (52 of 110) and we could preoperatively detect 46% (24 of 52) of node-positive patients.

## DISCUSSION

It is well documented that axillary nodal status is a major prognostic indicator and a guide to the need for adjuvant therapy. Traditionally, axillary dissection is performed primarily to obtain information on the histological nodal status of the patient and therapeutically to reduce the risk of axillary recurrence. Complete axillary dissection causes morbidity and patients who are node negative derive no benefit from it. Our study shows that if nodal positivity can be determined preoperatively by imaging techniques and percutaneous biopsy, a group of patients who maximally benefit from axillary dissection can be identified. A knowledge of nodal status preoperatively, along with other factors such as histological grade on core biopsy, may also indicate the need for postoperative mastectomy flap irradiation, which would influence the feasibility of immediate reconstruction (O'Rourke *et al*, 1994). A preoperative knowledge of node positivity is seen by some as an indication for neo-adjuvant chemotherapy.

The imaging techniques that have been extensively investigated to assess the axilla preoperatively are US with a reported sensitivity of 56 – 72% and a specificity of 70 – 90% (Bruneton *et al*, 1986; de Freitas *et al*, 1991; Vaidya *et al*, 1996; Feu *et al*, 1997), scintimammography with a sensitivity of 67 – 100% and a specificity of 80 – 90% (Dupont *et al*, 2001; Lumachi *et al*, 2001), CT with a sensitivity of 90% and a specificity of 82% (March *et al*, 1991; Uematsu *et al*, 2001) and colour Doppler with a sensitivity of 76% (Yang *et al*, 2000, 2001). The sensitivity and specificity achieved using these techniques does not allow a reliable selection of patients for full axillary clearance or a more minimally invasive procedure. More recent advances such as dynamic contrast enhanced MRI has a sensitivity and an NPV of 100% (Yoshimura *et al*, 1999; Murray *et al*, 2002) and 2-[(18) F] fluoro-2-deoxy-D-glucose-PET has a sensitivity of 82 – 95% and an NPV of 88% (Greco *et al*, 2001; Wahl, 2001). These two imaging modalities may be reliable in predicting the absence of nodal metastases in women with breast cancer, but these techniques are not readily available, are expensive and the results need to be confirmed in large multicentre studies. Although some of the above techniques are less expensive and have higher sensitivity than US-guided core biopsy, their PPV is appreciably less than 100%. If surgical management were based on these tests, the number of women with axillary clearance who did not have nodal metastases would be higher. Although US core biopsy is much less sensitive in our hands, the PPV is 100% and this enables our surgeons to perform axillary clearance selectively as a primary procedure in a group of patients with proven metastases on core biopsy.

In many of the studies assessing the role of US in the detection of malignant nodes, a number of criteria such as size, presence or absence of nodal hilum, echo texture, shape, focal doubling of the cortical thickness and cortical morphology were used independently or in combination. However, the US study by Vassallo *et al* (1992) and a more recent *in vitro* study using high-resolution CT by Uematsu *et al* (2001) have shown that nodal shape, that is, a longitudinal by transverse axis ratio of less than 2, and abnormal morphology of the cortex with a thickness of more than 2 mm are the two most reliable criteria in predicting malignancy *in vivo*. We adopted these criteria in our study to identify abnormal nodes and selectively biopsy them. The ease of assessment of these factors made it feasible to assess the axilla at the same time as US of the suspected primary malignancy in the breast.

In order to increase the specificity of preoperative assessment of the axilla, a number of groups have performed US-guided FNA (Bonnema *et al*, 1997; Verbanck *et al*, 1997; De Kanter *et al*, 1999; Krishnamurthy *et al*, 2002). Bonnema and de Kanter used this technique to detect axillary metastases in patients with operable breast cancer and preoperatively identified 63% (39 of 62) and 36% (31 of 87) of node-positive patients, respectively.

It is well documented that core biopsy of breast lesions is superior to FNA. We therefore thought it likely that core biopsy might prove to be superior to FNA in evaluating axillary lymph nodes. We used core biopsy with a 14-gauge biopsy needle to obtain axillary node samples. Using this technique to biopsy nodes that fulfilled our criteria, we could detect 27 of 64 (42%) of node-positive patients preoperatively. In all, 65% of all ultrasonographically abnormal nodes in our study had axillary metastases. However, 29% of patients with no identifiable nodes and 22% with normal nodes also had metastases. The similarity of nodal positivity in the normal and no node seen group suggests that lowering the threshold for biopsy of nodes seen would not be that effective in increasing the preoperative diagnosis of axillary metastases. US and core biopsy of abnormal nodes was reliable in detecting nodal metastases when the axillary burden of disease was high (four or more involved nodes). However, this technique was not sensitive in detecting axillary metastases when the disease burden was low (three or fewer nodes involved). The experience of our unit and others shows that a sampling procedure combined with axillary radiotherapy for patients with one to three positive nodes provides excellent long-term disease control with minimal morbidity (Lambah *et al*, 2001; Rampaul *et al*, in press). Patients who benefit most from axillary clearance are those with four or more positive nodes. This study demonstrates how these patients can be identified preoperatively. In our experience, there was increasing incidence of axillary metastases and their detection by US-guided biopsy with increasing size and grade of the invasive cancer. Our results using core biopsies are similar to previous studies using US-guided FNA. It is unclear whether FNA or core is best and how many passes are required for optimal results. There have been reports that gamma probe and US-guided FNA might be a potentially useful method for preoperative detection of sentinel node metastases (Motomura *et al*, 2001). Perhaps the sensitivity of US-guided biopsy could be improved by increasing the number of passes and samples obtained from the visualised abnormal nodes, and by preferentially imaging the sentinel node by dual scanning with a gamma probe and high-frequency US.

In conclusion, US can identify abnormal lymph nodes in patients presenting with primary operable breast cancer. In total, 65% of these nodes are malignant and this can often be confirmed by US-guided core biopsy. In all, 42% of all node-positive patients were correctly identified preoperatively.
